# p62/IMP2 stimulates cell migration and reduces cell adhesion in breast cancer

**DOI:** 10.18632/oncotarget.5328

**Published:** 2015-09-23

**Authors:** Yang Li, Giulio Francia, Jian-Ying Zhang

**Affiliations:** ^1^ Department of Biological Sciences & NIH-Sponsored Border Biomedical Research Center, The University of Texas at El Paso, El Paso, TX 79968, USA

**Keywords:** breast cancer, IGF2 mRNA binding protein 2, cell migration, cell adhesion, extracellular matrix

## Abstract

p62/IMP2 is an oncofetal protein that is overexpressed in several types of cancer, and is a member of the family of insulin-like growth factor 2 mRNA binding proteins. We previously reported that high levels of p62/IMP2 autoantibody are present in sera from cancer patients, compared to healthy individuals. Here, we report the overexpression of p62/IMP2 in tumor tissues of 72 out of 104 cases of human breast cancer, and high levels of p62/IMP2 autoantibody in patients’ sera (in 63 out of 216 cases). To explore the role of p62/IMP2 in breast cancer progression, we generated p62/IMP2 transfected variants of two human breast cancer cell lines: MDA-MB-231 and LM2-4. Using *in vitro* assays we found that overexpression of p62/IMP2 can increase cell migration, and reduce cell adhesion to extracellular matrix (ECM) proteins. A Human Extracellular Matrix and Adhesion Molecules qPCR array was performed with our generated variants, and it identified a group of mRNAs whose expression was altered with p62/IMP2 overexpression, including connective tissue growth factor (CTGF) mRNA – which we show to be a p62/IMP2 binding partner. Overall, our results provide new insights into the molecular mechanism by which p62/IMP2 can contribute to breast cancer progression.

## INTRODUCTION

The insulin like growth factor 2 mRNA-binding protein (IMP) family is a group of proteins involved in fetal development [1]. Three members (IMP1, p62/IMP2, IMP3) have been reported, and they have similar protein structures, composed of two RNA recognition motif domains and four K homology domains [2]. The primary function of these proteins is to bind specific mRNAs, stabilize and extend their half-life. All IMPs have been reported to be overexpressed in various cancers, including breast cancer, lung cancer, colorectal adenocarcinoma, and melanoma [3].

The IGF2 mRNA-binding protein 2 (p62/IMP2) remains the least-studied member of the IMP family [4]. p62/IMP2 is regulated by high-mobility group AT-hook 2 protein (HMGA2). HMGA2 can bind to an AT-rich region in the first intron of p62/IMP2 and regulate p62/IMP2 transcription [5]. As an mRNA-binding protein, p62/IMP2 can target different mRNAs. Among p62/IMP2 target mRNAs, most are associated with cancer progression, such as IGF2 mRNA and c-myc mRNA [3]. A number of p62/IMP2 target mRNAs have been implicated in the growth, migration, adhesion, or in the energy metabolism of cancer cells [4, 6–8].

We originally reported p62/IMP2 as a tumor-associated antigen (TAA) in hepatocellular carcinoma [9]. A high frequency of its autoantibody can be observed in several types of cancer, as we previously reported [10–12]. Aside from the presence of its autoantibody, the p62/IMP2 protein is overexpressed in several tumor types, including liposarcomas, hepatocellular carcinomas (HCC), and endometrial adenocarcinomas [5, 13, 14]. Overexpression of p62/IMP2 was also observed in some tumor cell lines (HepG2, SNU449, H1299, U2OS, HT-29, PANC-1, and IF6) [3]. However, there have been few studies on p62/IMP2 expression in breast cancer. In contrast, IMP1, a member of the IMP family with 84% sequence similarity to p62/IMP2 [15], has been reported as being amplified (in gene copy number) in breast cancer [16]; IMP1 was also reported to be overexpressed in mammary epithelial cells of adult transgenic mice that show high incidence of breast cancer development [17]. In this study we evaluated whether p62/IMP2 is similarly overexpressed in breast cancer progression. We found that p62/IMP2 was overexpressed in breast tumor tissues, and high levels of its autoantibody were present in the sera of a number of patients with breast cancer, compared to healthy individuals. By transfection into breast cancer cell lines, we also found that overexpression of p62/IMP2 in breast cancer cells can increase cell migration and reduce cell adhesion.

## RESULTS

### p62/IMP2 is overexpressed in human breast cancer tissues

**Figure 1:**
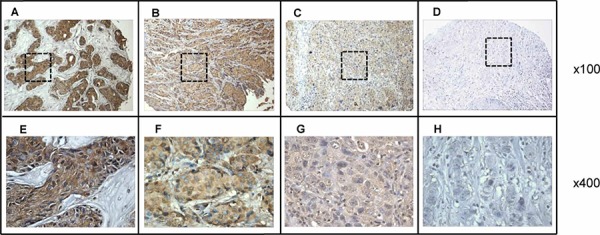
Immunohistochemical analysis of p62/IMP2 in four representative breast tumor tissues **A.** breast tumor tissue with high expression of p62/IMP2; **B.** breast tumor tissue with moderate expression of p62/IMP2; **C.** breast tumor tissue with weak expression of p62/IMP2; **D.** breast tumor tissue with negative expression of p62/IMP2 (Magnification: ×100). **E, F, G.** and **H.** The corresponding area (rectangle) of A, B, C, and D were enlarged (and presented below; magnification: ×400). Tissue slides were stained with anti-p62/IMP2 antibody at a 1:500 dilution.

We performed immunohistochemistry (IHC) analysis of p62/IMP2 on 118 breast tissues, including 104 tumor tissues and 14 adjacent normal tissues (Table 1 shows a summary of the data obtained). High expression of p62/IMP2 was observed in human breast tumor tissues (72/104 cases, corresponding to 69%), and low expression of p62/IMP2 was observed in the adjacent normal tissues (2/14 cases, corresponding to 14%). Figure 1 shows 4 representative examples of p62/IMP2 staining observed using a 4-level scoring system.

**Table 1:** Expression of p62/IMP2 in breast cancer tissues and adjacent tissues

Type of Tissues	Positive staining			
	Total	+++	++	+
Tumor Tissues (104)	72/104 (69%)*	10 (14%)	23 (32%)	39 (54%)
Normal Tissues (14)	2/14 (14%)	0 (0%)	1 (7%)	1 (7%)

* *p* < 0.01, compared with normal group (Fisher's exact test).

A four-level scoring system was used to evaluate the staining intensity of p62/IMP2:

(−) = negative expression, (+) = weak expression level, (++) = moderate expression level, (+++) = high expression level.

**Figure 2:**
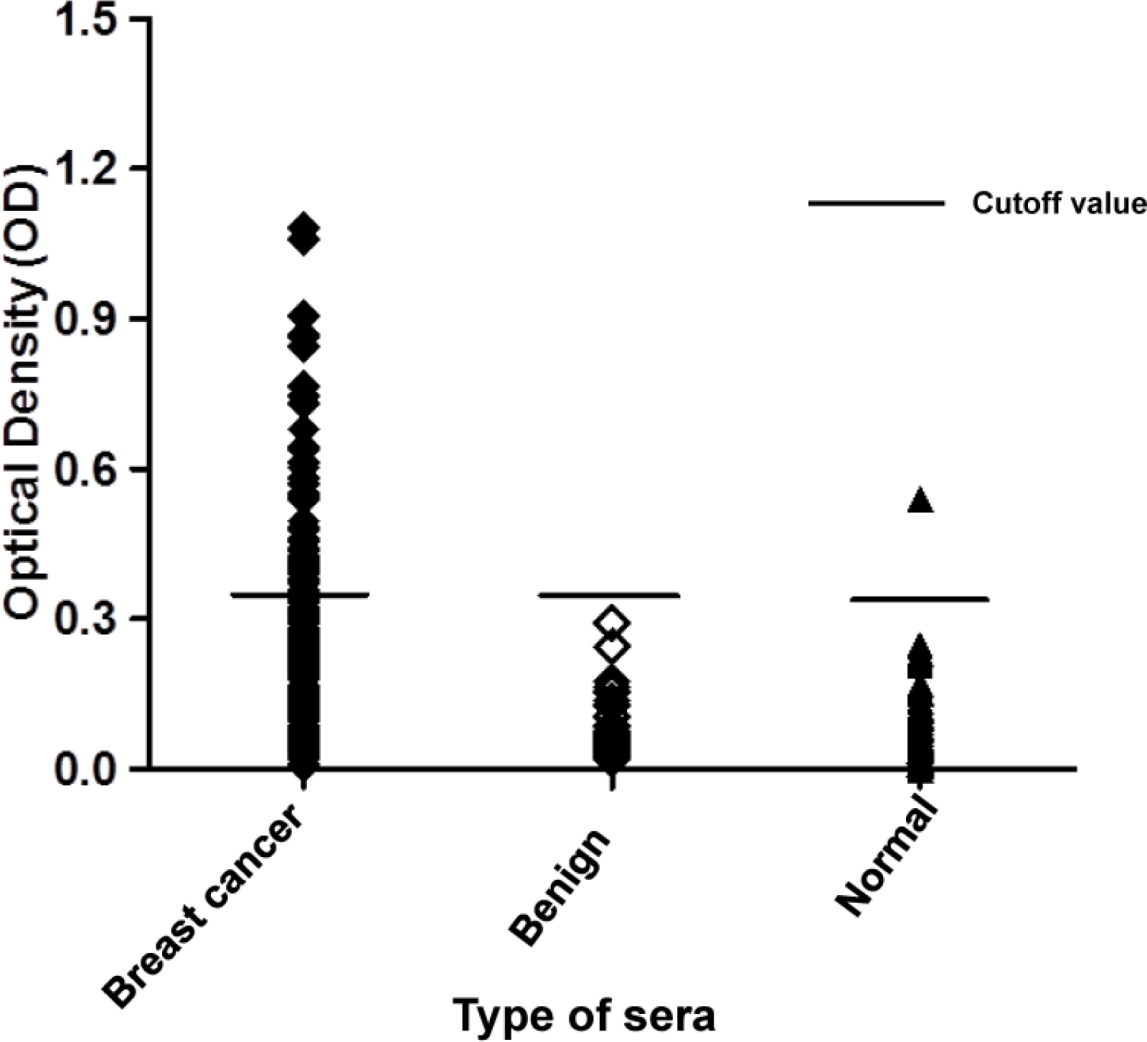
Titers of autoantibody against p62/IMP2 in sera from patients with breast cancer, or from individuals with benign lumps, and normal human sera In the graph, titer of anti-p62/IMP2 autoantibody in sera from patients with breast cancer was much higher than that in the sera from individuals with benign breast lumps, and that in sera from healthy individuals. The distribution of autoantibody titers is indicated as optical density (OD) obtained from ELISA. The mean + 3SD of normal human sera are shown in relationship to all serum samples.

p62/IMP2 has been described as a tumor-associated antigen, and a high frequency of detectable autoantibody to p62/IMP2 in sera from cancer patients has been reported. To detect the prevalence of p62/IMP2 autoantibody in sera from patients with breast cancer, recombinant p62/IMP2 protein was used as the coating antigen in an Enzyme-linked immunosorbent assay (ELISA) to screen sera from breast cancer patients and from patients with benign breast lumps, as well as in sera from normal individuals. Analysis of the sera (Table 2) from 216 patients with breast cancer showed that the positive frequency of detectable p62/IMP2 autoantibody was 29% (63/216), which was significantly higher than that in sera from normal individuals (1%, 1/73), and from patients with benign lumps (0%, 0/34). From the distribution of optical density (OD) value from readings of the three groups (Figure 2), it was evident that the levels in the sera from most of patients with breast cancer were above the cutoff OD value (0.32), indicating that these sera were positive for p62/IMP2 autoantibody. In contrast, only one serum sample from a normal individual showed anti-p62/IMP2 autoantibody (OD: 0.54), and no sera from patients with benign breast lumps produced OD reading above the cutoff value.

**Table 2:** Frequency of Autoantibody Response to p62/IMP2 Protein

Group	Total	Positive (%)
Breast cancer	216	63 (29%)*^#^
Benign	34	0 (0%)
Normal	73	1 (1%)
Total	323	64

Cutoff value: Mean+3SD of normal group;

Breast cancer group vs Normal group: *p < 0.01;

Breast cancer group vs Benign group: ^#^*p* < 0.01

Breast cancer: sera from patients with breast cancer

Benign: sera from patients with breast benign lumps

Normal: sera from normal individuals

### Overexpression of p62/IMP2 contributes to overexpression of c-myc in the MDA-MB-231 cells and LM2-4 cells

**Figure 3:**
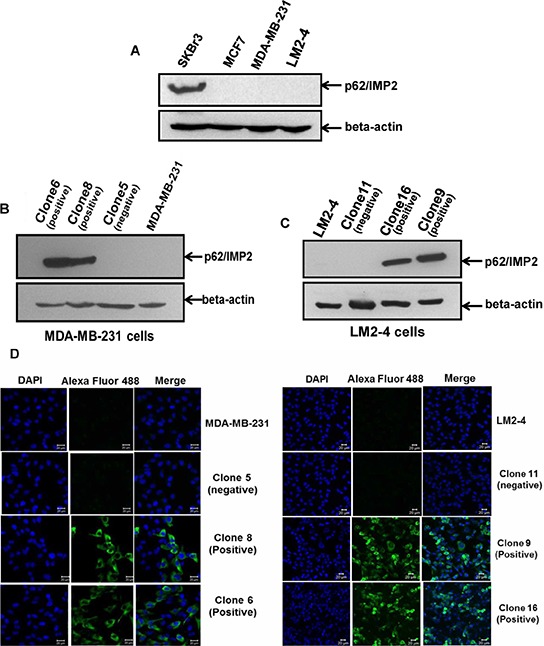
The generation of variants with stable overexpression of p62/IMP2 in breast cancer cells Western blotting analysis of p62/IMP2 in four human breast cancer cell lines showed p62/IMP2 was expressed in SKBr3 cells **A.** Variants with stable overexpression of p62/IMP2 were generated with MDA-MB-231 cells **B.** and LM2-4 cells **C.** Western blotting analysis showed that two p62/IMP2–transfected clones (clone 6 & clone 8) of MDA-MB-231 cells and two p62/IMP2-transfected clones (clone 16 & clone 9) of LM2-4 cells strongly expressed the p62/IMP2 protein. These clones were chosen as p62/IMP2 positive clones. Two empty vector-transfected clones (clone 5 for MDA-MB-231 cells and clone 11 for LM2-4 cells), that do not express p62/IMP2 protein, were chosen as p62/IMP2 negative clones. **D.** Immunofluorescence staining of p62/IMP2 in MDA-MB-231 cells and LM2-4 cells. There was no p62/IMP2 staining in the negative clones and in the parental cells. In the positive clones, the staining of p62/IMP2 (green) was mainly localized to the cytoplasm. Cell nucleuses were stained with DAPI (blue).

**Figure 4:**
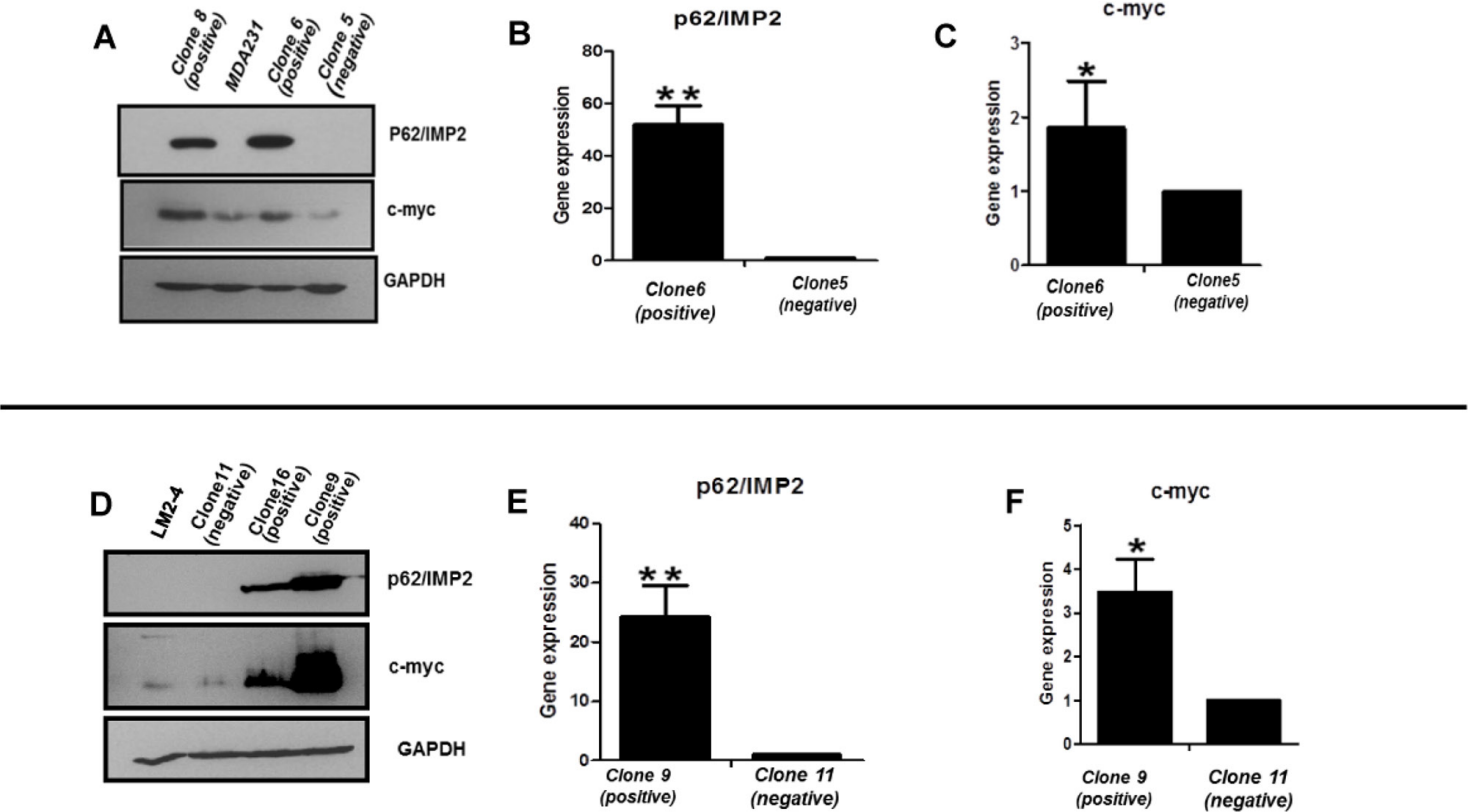
Expression of c-myc protein is increased with the overexpression of p62/IMP2 Increased c-myc protein was detected by Western blotting in MDA-MB-231 cells **A.** and LM2-4 cells **D.** transfected with p62/IMP2. The increased c-myc in mRNA level was detected by real-time PCR in MDA-MB-231 cells **B, C.** and LM2-4 cells **E, F.** that transfected with p62/IMP2. A *t*-test was applied to analyze the results. *p < 0.05 when compared with the negative clones. * *p < 0.01 when compared with negative clones. Values represent averages ± SEM of three independent measurements.

To investigate the biological role of p62/IMP2 in breast cancer progression, we screened a panel of breast cancer cell lines and found that p62/IMP2 was expressed in the SKBr3 cell line (Figure 3A). Next we generated, by plasmid transfection, p62/IMP2 overexpressing variants of the MDA-MB-231 and LM2-4 cell lines. Two p62/IMP2 positive clones and one p62/IMP2 negative clone were chosen respectively from the two cell lines (Figure 3B and Figure 3C). Indirect immunofluorescence results confirmed the Western blotting result from the selected clones, and p62/IMP2 was found to be detectable in the cytoplasm (Figure 3D). In our p62/IMP2 overexpressing variants, we also found that c-myc protein and c-myc in mRNA level were increased compared to controls (including negative clones, and parental cells) (Figure 4), which is consistent with previous reports [7].

### p62/IMP2 increases cell migration and reduces cell adhesion, but does not impact cell proliferation in breast cancer cells

**Figure 5:**
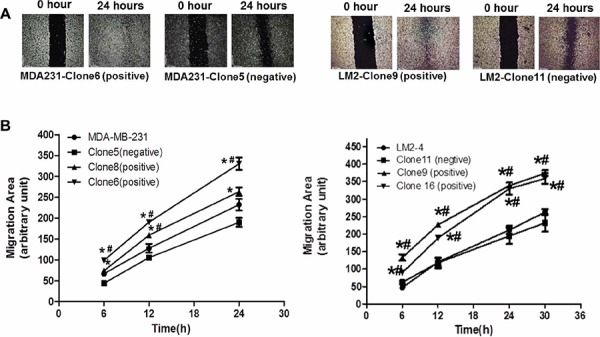

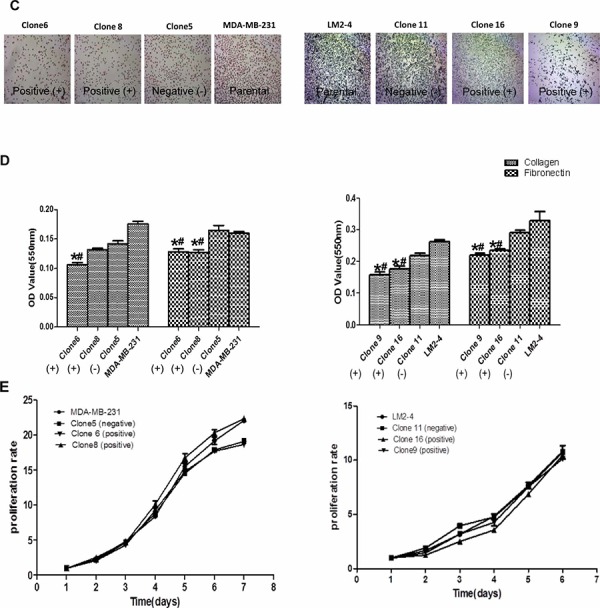
The impact of p62/IMP2 on breast cancer cells Overexpression of p62/IMP2 can increase cell migration of MDA-MB-231 cells and LM2-4 cells. Wound scratch images of p62/IMP2 positive/negative clones at different time points **A.** The level of cell migration into the wound scratch was quantified as migration area at each time point **B.** Overexpression of p62/IMP2 can reduce cell adhesion of MDA-MB-231 cells and LM2 cells. Crystal violet staining images of those p62/IMP2 positive/negative cells that attached at the bottom of collagen coated-wells **C.** The number of attached cells from different cell lines was presented as optical density values following the staining **D.** Growth curve of p62/IMP2 positive/negative cells were compared, and there were no significant changes between groups **E.** A One-way ANOVA was applied to analyze the results. *p < 0.05 when compared with negative clones. ^#^*p* < 0.05 when compared with parental cells. Values represent averages ± SEM of three independent measurements.

To determine the impact of p62/IMP2 on breast cancer cells, the generated p62/IMP2 transfected variants were used to perform *in vitro* assays to evaluate relative cell adhesion, cell migration, and cell proliferation, compared to controls. The results from the wound healing assay suggested an increased migration of p62/IMP2 positive cells compared to controls (Figure 5A). Thus, overexpression of p62/IMP2 in breast cancer cells increased ‘wound’ closure by 50% to 70% (Figure 5B). We also tested cell adhesion to collagen, and to fibronectin. We found that p62/IMP2 positive cells were less adherent compared to control cells (Figure 5C). Thus overexpression of p62/IMP2 reduced cell adhesion by 30%-50% (Figure 5D). Relative cell growth was also examined using a proliferation assay, but no impact of p62/IMP2 expression on breast cancer cell proliferation was observed over a 6–7 day monitoring period (Figure 5E). Taken together, these data indicate that p62/IMP2 increases cell migration and reduces cell adhesion in breast cancer cells *in vitro*.

### Selective gene expression profiling of human breast cancer cells overexpressing p62/IMP2

**Figure 6:**
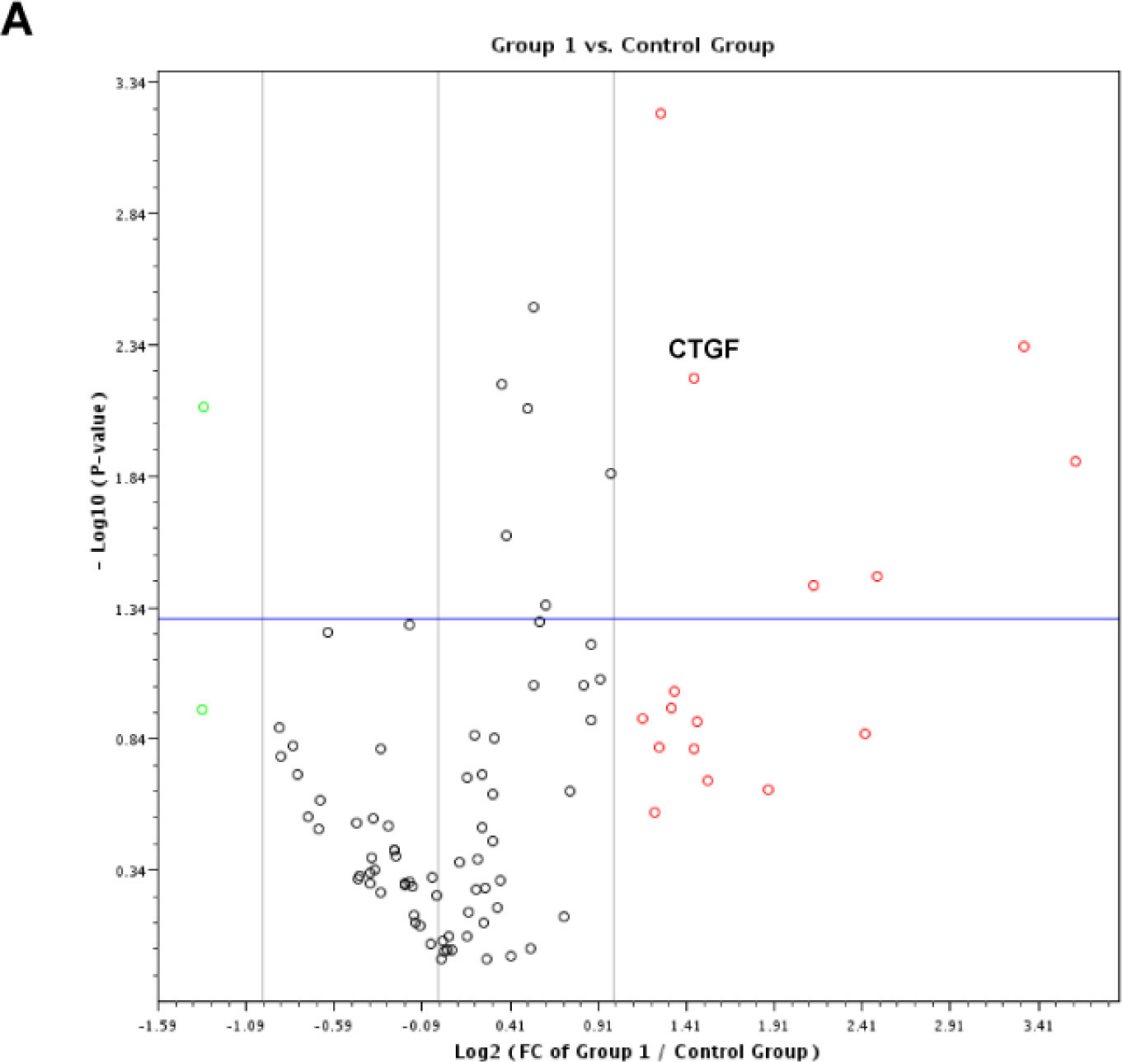

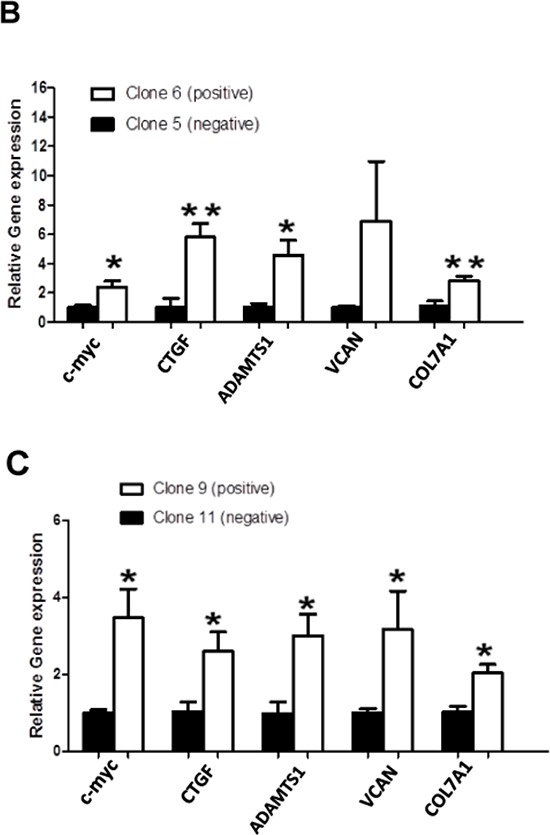
Changes in genes expression associated with p62/IMP2 overexpression A volcano plot for the relative gene expression from MDA-MB-231 cells with overexpression of p62/IMP2, using Human Extracellular Matrix & Adhesion Molecules RT2 Profiler PCR Array **A.** Down-regulated genes are highlighted as green, and up-regulated genes in red (Fold difference: 2). Gene expression above the horizontal line indicated that *p*-value less than, or equal to, 0.05. Group 1: clone 6 (p62/IMP2+) & Control Group: clone 5 (p62/IMP2-). Increased mRNA of versican, CTGF, ADAMTS1, and COL7A1 were confirmed again with our primers in MDA-MB-231 cells. **B.** and LM2-4 cells **C.** qPCR data were normalized to the housekeeping gene, GAPDH. A *t*-test was applied to analyze the results. *p < 0.05 when compared with the negative clones. * *p < 0.01 when compared with negative clones. Values represent averages ± SEM of three independent measurements.

To better understand how p62/IMP2 regulates cell migration and cell adhesion in breast cancer cells, a commercially available Human Extracellular Matrix & Adhesion Molecules RT^2^ Profiler PCR Array was used to compare our p62/IMP2 transfectants with the p62/IMP2 negative clone cells. This array profiles the expression of 84 mRNA of genes involved in cell-cell and cell-matrix interactions. Out of 84 genes, 16 were found to be up-regulated in 2-fold or greater in p62/IMP2 overexpressing cells, whereas only 2 genes were found to be down-regulated (Figure 6A). At last 4 genes in this array (Table 3), including connective tissue growth factor (CTGF), versican (VCAN), ADAM metallopeptidase with thrombospondin type 1 motif, 1 (ADAMTS1), and collagen, type VII, alpha 1 (COL7A1) were confirmed, by RT-PCR, in variants from MDA-MB-231 and LM2-4 cell lines in our additional experiment (Figure 6B and Figure 6C).

**Table 3:** The changed genes encoding extracellular matrix and adhesion molecules with stable overexpression of p62/IMP2 in MDA-MB-231 cells (also confirmed in LM2-4 cells)

Gene	Description	Fold Regulation	GenBank	Function
VCAN	Versican	12.3084	NM_004385	An anti-adhesive molecule
CTGF	Connective tissue growth factor	2.735	NM_001901	Tissue wound repair
ADAMTS1	ADAM metallopeptidase with thrombospondin type 1 motif, 1	4.3815	NM_006988	Tumor growth and metastasis
COL7AL	Collagen, type VII, alpha1	2.5052	NM_000094	Interaction with laminin and fibronectin

### p62/IMP2 binds to and increases the stability of CTGF mRNA

**Figure 7:**
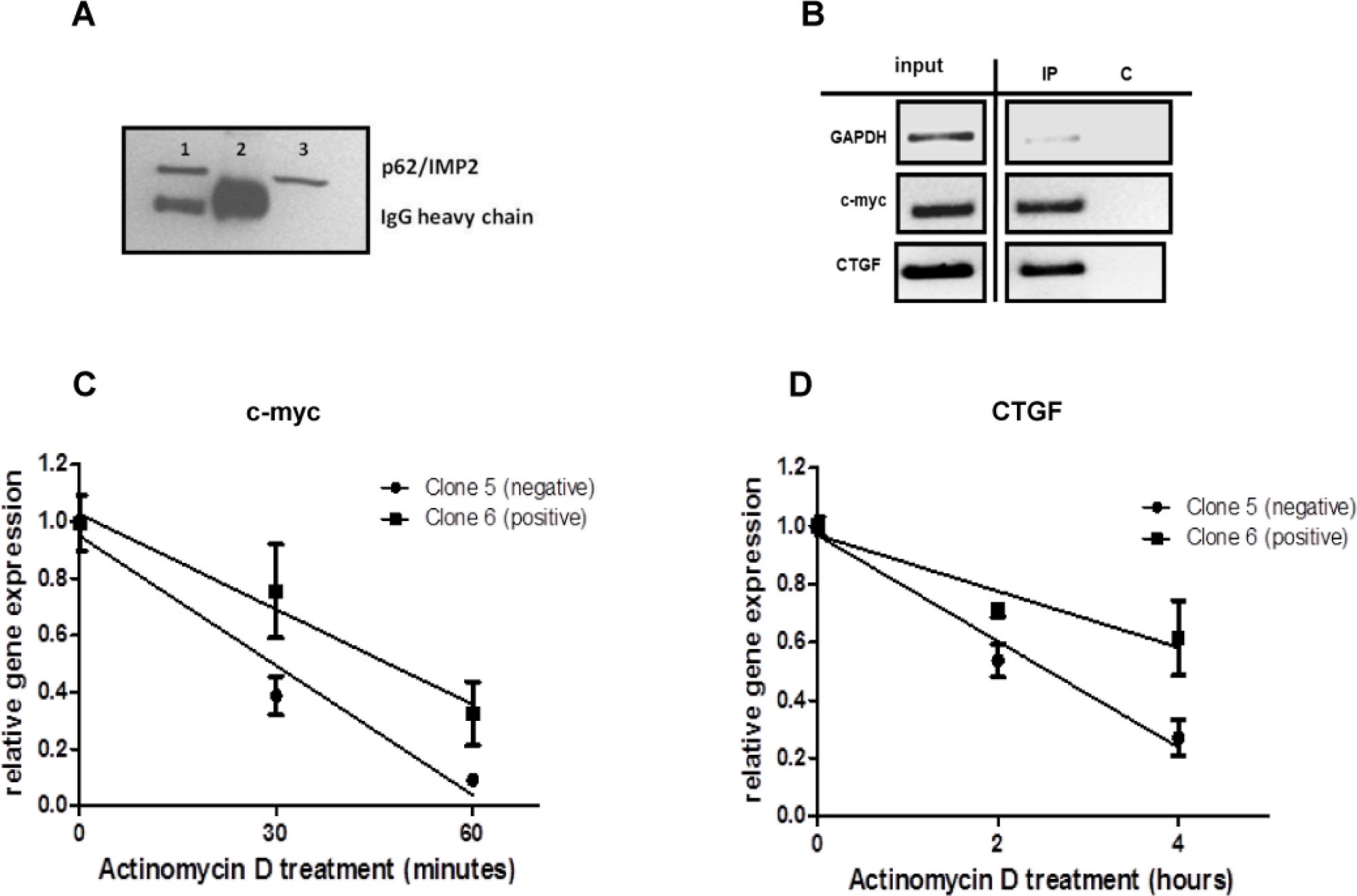
CTGF mRNA is a novel target of p62/IMP2 RIP assay was performed in MDA-MB-231 cells with overexpression of p62/IMP2 **A & B.** (A) p62/IMP2 expression level was analyzed by western blotting. Lane1: post-IP beads of anti-p62/IMP2 antibody. Lane2: post-IP beads of Normal mouse IgG. Lane3: input sample. (B) p62/IMP2 binds to mRNAs of CTGF and c-myc but not GAPDH. **C.** p62/IMP2 regulates the stability of c-myc mRNA. **D.** p62/IMP2 regulates stability of CTGF mRNA.

The CTGF protein is involved in the process of wound repair [18]. CTGF expression can be regulated by microRNAs that interact with its 3′-UTR, which results in repressed translation of CTGF or the degradation of its transcripts [19]. Previous studies have demonstrated that p62/IMP2 is an mRNA-binding protein, which can bind its target mRNAs in the 5′ or 3′ untranslated regions (UTRs). Therefore we hypothesized that CTGF may be a novel target of p62/IMP2 in breast cancer cells. The results from Figure 7A and Figure 7B show that CTGF mRNA can be immunoprecipitated with p62/IMP2, but not with GAPDH, by using p62/IMP2 antibody. The results in Figure 7D demonstrate that this binding can stabilize the mRNA of CTGF. In clone 5 (p62/IMP2 negative) of MDA-MB-231 cells, the half-life of CTGF mRNA was 2.2 ± 0.03 hour. In contrast, the mRNA half-life of CTGF was increased 2.3-fold to 5.1 ± 0.02 hours in clone 6 (p62/IMP2 positive) of MDA-MB-231 cells (*p* < 0.01). c-myc mRNA, which is a well-studied target of p62/IMP2, was used as a positive control (Figure 7B and Figure 7C). The half-life of c-myc mRNA was 0.3 ± 0.19 hours in clone 5, and the half-life of c-myc mRNA in clone 6 was 0.8 ± 0.23 hours (*p* = 0.044). In summary, our data indicated that p62/IMP2 can bind to and stabilize the mRNA of CTGF.

## DISCUSSION

The results of this study indicate that p62/IMP2 overexpression is observed in breast cancer tissues. Furthermore, by ELISA we observed a high frequency of anti-p62/IMP2 autoantibody in sera from breast cancer patients. A previous study demonstrated that p62/IMP2 is developmentally regulated and is expressed in fetal liver tissues but not in adult livers, and yet it is also overexpressed in liver tissues from patients with HCC [13]. Detectable autoantibody to p62/IMP2 was found to be present in 21.1% of HCC patients from China but not in patients with precursor conditions such as chronic hepatitis and liver cirrhosis [9]. Liu et al. found the overexpression of p62/IMP2 in colon cancer tissues by IHC [10]. In a study of 82 patients with digestive canal tumors, 38.6% of patients were positive for autoantibody to p62/IMP2, and 33.7% of those patients had metastatic disease, which suggests that the anti-p62/IMP2 autoantibody might be a predictive marker for cancer metastasis [20]. Overall these data indicate a role of p62/IMP2 in various human tumor types. Anti-TAA autoantibodies are stable and can readily be detected by using ELISA, which can make these autoantibodies as potential markers of diseases. And number of anti-TAA autoantibodies have been reported to be present in sera from cancer patients [21]. Furthermore, the elevation of anti-TAA autoantibody levels also correlates with the occurrence of some cancers, so the detection of the anti-TAA autoantibody can be used for the immunodiagnosis of some types of cancer [9, 11].

Furthermore, using p62/IMP2 transfected cells, we observed that p62/IMP2 can increase cell migration and reduce cell adhesion to the extracellular matrix. Since increased cell migration and reduced cell adhesion are important in the process of tumor cell invasion, and tumor spread [22, 23], our results suggest some mechanism by which p62/IMP2 overexpression may contribute to breast cancer progression. To uncover the mechanisms by which p62/IMP2 regulates cell migration and cell adhesion, a Human Extracellular Matrix & Adhesion Molecules RT^2^ Profiler PCR Array was carried out to observe mRNA changes regulated by p62/IMP2. We found that most of genes with altered expression were up-regulated by overexpression of p62/IMP2. Our possible explanation is that, in p62/IMP2 overexprssing cells, these mRNAs are stabilized by binding to the p62/IMP2 protein. Among these genes, CTGF mRNA which has a short half-life was chosen to test such a possibility. Our results indicated that CTGF mRNA is a target of p62/IMP2, as shown by the mRNA half-life assay and RIP assay. The functional features of CTGF are very similar to p62/IMP2. CTGF plays important roles in many biological processes, including cell migration and adhesion, tissue development and stem cell pluripotency [18, 24]. CTGF is also shown involved in some human disorders, such as diabetes, and some types of cancer. Several studies showed that the up-regulation of CTGF correlates with cancer progression and metastasis such as in osteolytic metastasis of breast cancer [25, 26]. CTGF has been reported to promote tumor metastasis by increasing matrix metalloproteinase expression [27]. In a recent study, CTGF was shown to be up-regulated in p62/IMP2 transgenic mice and the up-regulated expression of CTGF is TGF-beta-independent [28]. This study also suggested that IL13 might be involved in its up-regulation. In that respect, our data showed that p62/IMP2 can bind to mRNA of CTGF directly and stabilize its mRNA.

How p62/IMP2 regulates other genes (such as ADAMTS1, VCAN and COL7A1) remains unknown. ADAMTS1 is necessary for normal growth, and ADAMTS1 null mice were observed to exhibit growth retardation [29, 30]. Versican is well-known as an anti-adhesive molecule, which has been reported to reduce the attachment of prostate cancer cells and melanoma to fibronectin-coated surfaces *in vitro* [31]. Therefore p62/IMP2 interacts with a number of genes associated with cell adhesion.

In summary, we observed that p62/IMP2 is highly expressed in breast cancer tissues. We also found that the overexpression of p62/IMP2 can regulate the mRNAs for extracellular matrix and adhesion molecules such as CTGF, ADAMTS1, COL7A1, and VCAN. These changes could contribute to the observed increased cell migration and reduced cell adhesion, which can contribute to an increased malignant behavior of breast cancer cells.

## MATERIALS AND METHODS

### Tissue and sera collection

For the analysis of breast cancer tissues and control tissues, formalin-fixed, paraffin-embedded, breast tissue arrays were purchased from US Biomax, Inc (MD, USA). These included (as described by US Biomax) 54 cases of breast invasive ductal carcinoma, 40 cases of lymph node metastasis, 10 cases of other types of breast cancer tissue, and 14 cases of adjacent normal tissue. A total of 323 human sera were analyzed, and they were obtained from the serum bank of The Cancer Autoimmunity and Epidemiology Research Laboratory at UTEP (University of Texas at El Paso). These samples were originally provided to us by our clinical collaborators. Among them, 216 serum samples were from patients diagnosed with breast cancer (70 samples from Germany, 104 samples from China, 42 samples from Mexico), 34 sera were from individuals with begin breast lumps, and 73 sera were from individuals with no evidence of malignancy. None of the patients with breast cancer received any chemotherapy or radiotherapy at the time whenthe sera were collected. All serum samples were collected from consenting individuals.

### Cell culture and transfection

MDA-MB-231 and LM2-4 cell lines were cultured in Dulbecco's Modified Eagle Medium (GIBCO, Life Techologies, Grand Island, NY, USA) with 10% fetal bovine serum (GIBCO, Life Technologies, Grand Island, NY, USA) and 100 units/ml penicillin plus 100 μg/ml streptomycin at 37°C with 10% CO2. Cells were plated in 6-well plates and transfected with Lipofectamine 2000 (Life Techologies, Grand Island, NY, USA). We used a p62-pcDNA3.1 plasmid to transfect MDA-MB-231 and LM2-4 cells, and pcDNA3.1 (empty vector) was used to generate control transfectants. Variants with stable overexpression of p62/IMP2 were selected in DMEM medium containing 1mg/ml G418.

### Enzyme-linked immunosorbent assay (ELISA)

ELISA was performed as described in our previous study [32]. In brief, polystyrene 96-wells microtiter plates (Thermo scientific, Waltham, MA, USA) were coated overnight at 4°C with purified recombinant p62/IMP2 (0.5 μg/ml) in phosphate-buffered saline (PBS), and then blocked with post-coating solution. Antigen-coated wells were incubated with 100 μl human sera, diluted at 1:100, for 2 hours. The antigen-antibody complex were further bound to HRP-conjugated goat anti-human IgG (Santa Cruz Biotechonolgy, Inc., Santa Cruz, CA, USA) and detected by the substrate 2,2′-azino-bis-3-ethylbenzo-thiazoline-6-sulfonic acid (ABTS: Sigma-Aldrich, St. Louis, MO, USA). Optical density (OD) was measured at 405 nm.

### Immunohistochemistry and immunofluorescence

Immunohistochemistry was performed using standard protocols. We used anti-p62/IMP2 antibody as primary antibody, and polyvalent biotinylated link was used as secondary antibody. DAB reagent was used for detection of p62/IMP2. For immunofluorescence, cells were seeded into 8-chamber culture slides. The cells were treated with p62/IMP2 antibody, and incubated with Alexa Flour 488 conjugate. Mounting medium containing DAPI was then added. Confocal fluorescence images were acquired with a laser scanning microscope (LSM 700; Zeiss, New York, NY, USA).

### *In vitro* wound healing assay

Cells were seeded in 6-well plates and cultured in DMEM medium with 2% FBS. A wound scratch was created using a sterile 1ml pipette tip. Scratch images of the wounds were photographed every 6 hours. The migration area of cells into the wound was quantified at each time point with Image J software.

### *In vitro* cell-extracellular matrix adhesion assay

96-well plates were coated with 10 μg/ml fibronectin or 10 μg/ml type I collagen, and incubated overnight at 4°C. Non-specific binding sites were blocked with 1% BSA in serum-free culture medium. Cells (5 × 10^3^) were cultured in the coated wells and incubated at 37°C for 1 to 2 hours. Then non-adherent cells were removed by washing with washing buffer (0.5% BSA in serum-free culture medium). Attached cells were stained with crystal violet (5 mg/ml in 20% ethanol). Optical density was measured at 550 nm.

### Cell proliferation assay

Cells (5 × 10^3^) were seeded on to 96-well plates, grown for 1 to 7 days. The growth of cells was determined by the sulforhodamine (SRB) assay. Briefly, the cells were fixed with 10% trichloracetic acid (TCA) for 1 hour at 4°C, rinsed five times with water, and air dried. Fixed cells were stained with 0.4% SRB in acetic acid. Unbound dye was removed by washing 5 times with 1% acetic acid, and plates were air dried. The staining was solubilized with 10 Mm Tris Base. The colorimetric reading was carried out in a microplate autoreader set at 515 nm.

### RNA isolation and quantitative real-time RT-PCR

Total RNA from breast cancer cells was isolated by AllPrep DNA/RNA/Protein Mini Kit (QIAGEN, Valencia, CA, USA). RNA integrity was assessed by 0.7% agarose gel electrophoresis. RNA was reversely transcribed with GoScriptTM Reverse Tanscription System. The following primers were used: c-myc forward, 5′-AG CGACTCTGAGGAGGAAC-3′ and reverse, 5′-CGT AGTTGTGCTGATGTGTG-3′; CTGF forward, 5′-CACAG AGTGGAGCGCCTGTTC-3′ and reverse, 5′-GATGCA CTTTTTGCCCTTCTTAATG-3′; ADAMTS1 forward, 5′-TG TGGTGTTTGCGGGGGAAATG-3′ and reverse, 5′-TCGA TGTTGGTGGCTCCAGTT-3′; COL7A1 forward, 5′-GGC TGCAATTCTCCATGTGG-3′ and reverse, 5′-CTGTGA GGCAACTCGCTTCA-3′; VCAN forward, 5′-TCACTCT AATCCCTGTCGTAATG-3′ and reverse, 5′-ATGTCTCGG TATCTTGCTCAC-3′; p62/IMP2 forward, 5′-AGTGGAAT TGCATGGGAAAATCA-3′ and reverse, 5′-CAACGGCG GTTTCTGTGTC-3′; GAPDH forward, 5′-GAAGGTG AAGGTCGGAGTC-3′ and reverse, 5′-GAAGATGGTG ATGGGATTTC-3′. Real-time PCR was performed with MyiQ Single Color Real-time PCR Detection System, as was the RT Profiler PCR Array kit.

### RNA immunoprecipitation

p62/IMP2-bound RIP was performed according to the RiboCluster Profiler RIP-Assay Kit (Medical & Biological Laboratories CO., LTD. Japan). Briefly, cell lysates were collected from p62/IMP2 positive cells, and then the whole complexes of p62/IMP2-targets from cell lysate were pulled down with p62/IMP2 antibody. The target mRNAs were separated and isolated for subsequent reverse transcription.

### mRNA half-life analysis

Cells (1 × 10^6^) seeded in to 6-well plates were treated with 5ug/ml actinomycin D (Sigma, St. Louis, MO, USA), and then these cells were collected every 30 minutes or 2 hours. Total RNA was isolated from collected cells. The amount of GAPDH, c-myc and CTGF mRNAs was quantified by Real-Time RT-PCR.

### Statistical analysis

The mean OD value of the group of patients’ sera was compared using the Mann-Whitney *U* test. The frequency of antibody against p62/IMP2 in the sera from groups was compared using *X*^2^ test. The p62/IMP2 expression in breast tissues was analyzed by *X*^2^ test and Fisher's text. A one-way ANOVA was applied to analyze results from wound healing assay, adhesion assay, and proliferation assay. The mRNA half-life was calculated from the first-order decay constants (K) obtained with the PRISM software program, version 3.03 (GraphPad, San Diego, CA): half-life = ln2/k. Comparisons between half-life of mRNAs were performed with the *t*-test. Data were presented as means ± SEM of three independent experiments. *p* < 0.05 was considered statistically significant.
